# Borehole Optical Fibre Distributed Temperature Sensing vs. Manual Temperature Logging for Geothermal Condition Assessment: Results of the OptiSGE Project

**DOI:** 10.3390/s24237419

**Published:** 2024-11-21

**Authors:** Maciej R. Kłonowski, Anders Nermoen, Peter J. Thomas, Urszula Wyrwalska, Weronika Pratkowiecka, Agnieszka Ładocha, Kirsti Midttømme, Paweł Brytan, Anna Krzonkalla, Adrianna Maćko, Karol Zawistowski, Jolanta Duczmańska-Kłonowska

**Affiliations:** 1 Polish Geological Institute-National Research Institute, Lower Silesian Branch, 53-122 Wrocław, Poland; urszula.wyrwalska@pgi.gov.pl (U.W.); weronika.pratkowiecka@pgi.gov.pl (W.P.); agnieszka.ladocha@pgi.gov.pl (A.Ł.); pawel.brytan@pgi.gov.pl (P.B.); anna.krzonkalla@pgi.gov.pl (A.K.); adrianna.macko@pgi.gov.pl (A.M.); karol.zawistowski@pgi.gov.pl (K.Z.); jolanta.duczmanska-klonowska@pgi.gov.pl (J.D.-K.); 2 NORCE Norwegian Research Centre, Nygårdsgaten 112, 5008 Bergen, Norway; aner@norceresearch.no (A.N.); peth@norceresearch.no (P.J.T.)

**Keywords:** subsurface, geothermal conditions, geological boreholes, optical fibre distributed temperature sensing (OF DTS), manual temperature logging, south-western Poland

## Abstract

Geothermal energy is a crucial component contributing to the development of local thermal energy systems as a carbon-neutral and reliable energy source. Insights into its availability derive from knowledge of geology, hydrogeology and the thermal regime of the subsurface. This expertise helps to locate and monitor geothermal installations as well as observe diverse aspects of natural and man-made thermal effects. Temperature measurements were performed in hydrogeological boreholes in south-western Poland using two methods, i.e., manual temperature logging and optical fibre distributed temperature sensing (OF DTS). It was assumed the water column in each borehole was under thermodynamic equilibrium with the local geothermal gradient of the subsurface, meaning rocks and aquifers. Most of the acquired results show typical patterns, with the upper part of the log depending on altitude, weather and climate as well as on seasonal temperature changes. For deeper parts, the temperature normally increases depending on the local geothermal gradient. The temperature logs for some boreholes located in urban agglomerations showed anthropogenic influence caused by the presence of infrastructure, the urban heat island effect, post-mining activities, etc. The presented research methods are suitable for applications connected with studies crucial to selecting the locations of geothermal installations and to optimize their technical parameters. The observations also help to identify zones of intensified groundwater flow, groundwater inrush into wells, fractured and fissured zones and many others.

## 1. Introduction

Optical fibres provide direct in situ insights into the temperature profile along geological boreholes and geothermal installations. Combining OF DTS measurements with manual temperature logging provides for the generation of additional data and the possibility of the comparative analysis of the results. These data are normally used to calculate the geothermal parameters of the subsurface and may lead to the estimation of the vertical heat flow through the lithosphere (subsurface). It can also be used to identify the presence of any groundwater inrush into the borehole through hydraulically preferential groundwater flow paths and thermal anomalies from geogenic or anthropogenic heat sources. This type of information is often used to optimize the location and development of ground source heating and cooling systems, and if the boreholes were equipped with permanent OF DTS monitoring systems, one would be able to enhance the long-term ground source heat exchange during the operational phase.

Temperature logging is often performed in geological boreholes to study spatiotemporal changes within the subsurface at the local and regional scales [1]. In addition, the results of subsurface temperature measurements are also useful for observing climatic changes [2] as the heat source of the shallow temperature is dictated by the storage of atmospheric ambient temperature and solar heat [3,4]. Temperature data are also useful for characterizing aquifer dynamics and estimating the thermal properties of the subsurface [5,6]. Subsurface temperature reference data are crucial for the development of low-temperature geothermal potential and suitability maps, assessment of potential of geothermal energy and determination of the thermal influence of underground infrastructure. Having access to such heat maps is key to developing the local heating and cooling systems as a carbon-neutral and reliable energy source. Choosing where the optimal location of high- and low-temperature geothermal installations should be constructed first, during a potential roll-out phase, rely on both assessing the local geothermal resource potential combined with the top-side need for local heat. Monitoring temperature data through time may be used to directly estimate the dynamic performance along the borehole. This information may be further used to manage ground source heat as a reliable, renewable energy source to maintain its availability in the long-term, lasting even up to several decades. In future, such data may be especially useful when operational heat extraction is coupled directly with the fluctuating variability of renewable electricity sources during the decarbonization of the energy system [7,8,9,10].

The subsurface temperature can be measured in boreholes in several different ways, including rather simple manual logging as well as profiling with more sophisticated and precise geophysical tools. OF DTS is a novel technique used for the passive measurement of temperature along geological and hydrogeological boreholes. In Poland, this kind of study has been performed for the first time ever to asses geothermal regime of subsurface and calculated selected geothermal parameters within the framework of the present “Optimization of Shallow Geothermal Energy Resources for Green Transition (OptiSGE)” project; thus, this paper presents the results of pioneering research. The project involved reference data collection in 23 selected boreholes in south-western Poland. To assess the geothermal conditions of the subsurface, two research methods were applied: OF DTS and manual temperature logging. The main objective of this paper is to showcase the usefulness of both methods and compare the results.

The paper describes the research carried out under the terms of the OptiSGE project implemented between September 2023 and August 2024 under the terms of the Open Call for Bilateral Initiatives in the thematic area of the Green Transition between Poland and Norway of the Fund for Bilateral Relations EEA and Norway Grants 2014–2021 Poland. The financing source was the Fund for Bilateral Relations of the European Economic Area Financial Mechanism 2014–2021 and Norwegian Financial Mechanism 2014–2021. The OptiSGE project aimed at the enhancement of green transition by the implementation of innovative methods for evaluating where low-temperature geothermal energy installations should be developed and how geothermal resources should be optimally used to support the economy and society in Poland and Norway. It also endeavoured to determine common obstacles for the roll-out of these resources in both countries and provided solutions with respect to the expanding geothermal energy sector.

## 2. Materials and Methods

### 2.1. Study Area and Geological and Hydrogeological Settings

**Figure 1:**
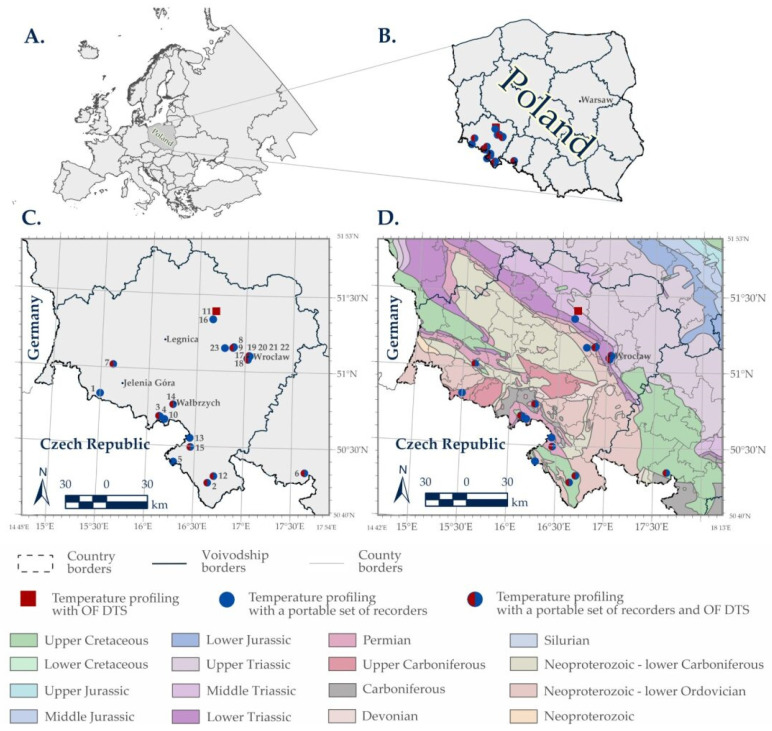
Contour maps of Europe (**A**) and Poland (**B**). Localization of the studied boreholes on the contour map (**C**) and simplified geological map (**D**) [25] of south-western Poland. Numbering of the studied boreholes according to Table 1.

The study area was south-western Poland, covering the entire region of Lower Silesia and part of the Opole region. Its southern part belongs to the Sudetes Mountains and its foreland, while the northern area is a part of the Silesian Lowlands [11]. The studied area and location of the boreholes are shown in Figure 1, while short characteristics of the boreholes are given in Table 1. Table 1 contains all necessary data such as borehole names and their CBDH ID numbers to identify the studied boreholes in the further parts of this paper. The research area belongs to two large and diverse geotectonic units: the Lower Silesian Block and the south-eastern part of the Fore-Sudetic Homocline. The former is a part of the Bohemian Massive. The Lower Silesian Block is divided into the Sudetic Block and the Fore-Sudetic Block, separated by two main faults, i.e., the Upper Elbe Fault Zone in the south-west and the Sudetic Marginal Fault in the north-east. These faults are the effect of the Cainozoic tectonic movements, which in the north caused the separation of the Fore-Sudetic Block by Middle Odra-Horst [12].

A characteristic feature of the geology of the Lower Silesian Block is its mosaic and varied structure caused by the complex geological evolution of the region through its entire period of formation, i.e., from the Upper Proterozoic to the Quaternary period [13]. The Sudetic Block is composed of igneous, metamorphic and sedimentary rocks and can be subdivided into several minor tectonic structures, including the North Sudetic Synclinorium and Intra-Sudetic Basin. Within the Fore-Sudetic Block, two major structural levels can be distinguished. The older one, made of metamorphic and igneous rocks, is partially covered by deposits forming a younger structural level [14,15].

The Fore-Sudetic Homocline is composed of Permian–Mesozoic rocks lying unconformably at an angle of several degrees on the folded Paleozoic basement [16]. Towards the south-east, the Permian deposits gradually thin out and Middle Jurassic, Middle and Upper Triassic deposits become wedged out as well. This rock complex consists mainly of terrestrial and marine sedimentary rocks with minor volcanic inserts and intrusions. The Permian bedrock contains some Paleozoic structures of the northern part of the Variscides of western Poland, including several fold units with considerable sizes [17]. Importantly, the Lower Permian sedimentary rocks host the biggest economic copper mineralization in Poland [18]. A simplified geological map of the studied area is presented in Figure 1D.

The hydrogeological conditions of the studied area result to a major extent from the local and regional geology. Fractured and fractured–porous aquifers prevail in the crystalline and sedimentary rocks of the Sudetes Mountains and their foreland, while porous ones are found in the Cainozoic cover on the Silesian Lowlands [19,20,21].

Due to the presence of fractures and fissures, as well as their opening degree, three vertical hydrogeological zones showing various hydrogeological and hydrogeochemical parameters can be distinguished within the crystalline bedrock. The shallowest zone is related to a relatively thin weathering cover including debris and the middle one is present within fissured and slightly weathered rocks, while the deepest one is associated with deep-seated faults and discontinuities [22]. Crystalline rocks are not considered to be very good aquifers and do not show a high ability to collect and transmit groundwater. The Cretaceous sandstone, limestone and marl forming porous-fissured and porous aquifers in the North Sudetic Synclinorium and Intra-Sudetic Synclinorium are more favourable in terms of hydrogeological settings [20,21].

Cainozoic aquifers are connected with glacial and fluvioglacial deposits with a high content of sand and gravel interbedded with less permeable till, clay and loam. The shallow and often unconfined Quaternary aquifers are present in contemporary river valleys and diverse glacial and periglacial geomorphological forms. Deeper Quaternary groundwater-bearing sediment often forms confined multi-layered aquifer systems created by the filling of buried valleys, i.e., sand and gravel interbedded with less permeable deposits. In some cases, finer Pleistocene sediments can be present at the bottom of the buried valleys. Neogene aquifers are mainly associated with sandy and gravelly layers, lenses and insertions within the thick clayey formation [20,21,23].

**Table 1:** Characteristics of the studied boreholes.

#	Borehole Name *	CBDH ID No. **	Altitude (m a.s.l.)	Drilling Year	Total Depth (m b.g.l.)	Land Use Type	Geotectonic Unit
1.	Czerwony Potok 1	8310064	723.30	2011	75.0	rural area/ forest	Izera-Karkonosze Massif
2.	Długopole Dolne 6R	9330025	355.60	1983	277.0	rural area/ pasture	Upper Nysa Graben
3.	Dobromyśl 1	8330199	504.84	2011	221.0	meadow	Intra-Sudetic Basin
4.	Dobromyśl 5B	8330178	531.30	n.a.	171.0	rural area/ forest	Intra-Sudetic Basin
5.	Krzyżanów 2	9000064	484.90	1987	300.0	rural area/ pasture	Orlica-Śnieżnik Dome
6.	Lubrza VA	9050125	248.50	1986	201.0	rural area/ arable land	Kędzierzyn Graben
7.	Łupki 1	7580052	274.90	1986	445.0	rural area/meadow	North Sudetic Synclinorium
8.	Marszowice osiedle	7630307	117.80	1984	135.0	suburban area/ low-rise dev.	Fore-Sudetic Homocline
9.	Marszowice pole	7630154	115.00	1976	131.5	suburban area/ meadow	Fore-Sudetic Homocline
10.	Mieroszów P2	8330135	499.20	1982	120.0	rural area/meadow	Intra-Sudetic Basin
11.	Pełczyn IVP	6890081	108.50	1982	517.0	rural area/meadow	Intra-Sudetic Basin
12.	Stary Waliszów 7R	9330028	385.00	1986	625.0	rural area/meadow	Intra-Sudetic Basin
13.	Tłumaczów 21N	8670005	350.50	1990	110.0	rural area/meadow	Intra-Sudetic Basin
14.	Wałbrzych Stara Kopalnia	n.a.	440.90	n.a.	n.a.	urban area/ post-industrial	Intra-Sudetic Basin
15.	Wambierzyce 18N	9000067	363.70	1988	500.0	rural area/meadow	Intra-Sudetic Basin
16.	Wołów 6	6890064	108.00	1979	200.0	rural area/meadow	Fore-Sudetic Homocline
17.	Wrocław Bastion Sakwowy	7640406	120.70	1965	115.00	urban area/ green area	Fore-Sudetic Homocline
18.	Wrocław Klasztor Bonifratów	7641078	117.60	1980	112.0	urban area/ built-up area	Fore-Sudetic Homocline
19.	Wrocław Leśnica 1A	7630374	122.60	n.a.	135.0	suburban area/ green area	Fore-Sudetic Homocline
20.	Wrocław Leśnica 3A	7630256	122.70	1981	250.0	suburban area/ green area	Fore-Sudetic Homocline
21.	Wrocław Szpital Kolejowy	7640365	124.30	1964	108.50	urban area/ built-up area	Fore-Sudetic Homocline
22.	Wrocław W-1	7641997	124.98	2018	90.00	urban area/ green area	Fore-Sudetic Homocline
23.	Wróblowice	7630295	142.50	1983	76.00	rural area	Fore-Sudetic Homocline

* In alphabetical order; ** CBDH—unique ID no. in the HYDRO Bank [24] database; n.a.—not available.

### 2.2. Characteristics of the Studied Boreholes

Some boreholes were excluded from the research due to the dimensions of their upper aperture being too small, resulting in the limited manoeuvrability of equipment. All 23 boreholes (Table 1) selected for further analysis are observation wells and piezometers filled with groundwater. All of them represent the major geotectonic units of the studied area as well as the main hydrogeological settings, including porous, fissured and fissured–porous aquifers. The wells were drilled between 1964 and 2018 and are located at an altitude varying from 108.0 to 723.3 m above sea level (m a.s.l.). Their total measured depth ranged from 75.0 to 625.0 m below ground level (m b.g.l.). The boreholes differ in terms of technical construction, as they normally have steel casing and screens, but in some cases, usually found in newer boreholes, the casing is made of hard PVC. The deeper parts of the older boreholes were drilled in harder rocks, i.e., sandstone, limestone, marl and crystalline rock, which were sometimes uncased, which in some cases led to wellbore wall collapse.

Depending on their location, 10 of the shallower boreholes, with a total measured depth of 76.0 to 200.0 m b.g.l., penetrate the subsurface to Neogene sediments. The deeper boreholes usually penetrate older rocks such as Cretaceous, Tertiary, Permian and Carboniferous sediments or crystalline bedrock of the Proterozoic age. In total, 9 of the boreholes are located in cities, i.e., in Wrocław and in Wałbrzych, while the remaining 14 are in smaller towns and villages, or outside built-up areas. The land use type of their location varies, covering both natural and man-made forms, i.e., forest, meadow, pasture, arable land, green areas, rural development, urban development and post-industrial areas.

The studied boreholes were not equipped with pumping units such as submersible pumps, tubing or electric cables or other infrastructure that could complicate the placement of the optical fibre and influence measurement results. Some boreholes, though, were equipped with automatic devices to register the groundwater table position. These were always removed at the time of the performed measurements. All the borehole data used in this research come from the HYDRO Bank [24] database run by the Polish Geological Institute–National Research Institute. The characteristics of the studied boreholes are shown in Table 1.

### 2.3. Manual Temperature Logging

Manual temperature logging was performed with a portable set of two recorders (Solinst Canada Ltd., Georgtown, ON, Canada) between September 2023 and November 2023. Both devices, a Levelogger 5 and Barologger 5 3001 (Table 2), were equipped with temperature and pressure sensors. The former is normally used for downhole measurements, while the latter is used to measure atmospheric temperature and pressure at the surface. The sensors were calibrated by the producer; thus, calibration before the measurement series was not required. The software Levelogger 4.6.3. enables the programming of the recorders, including the sampling frequency and barometric compensation of measurement records for both recorders [26].

**Figure 2:**
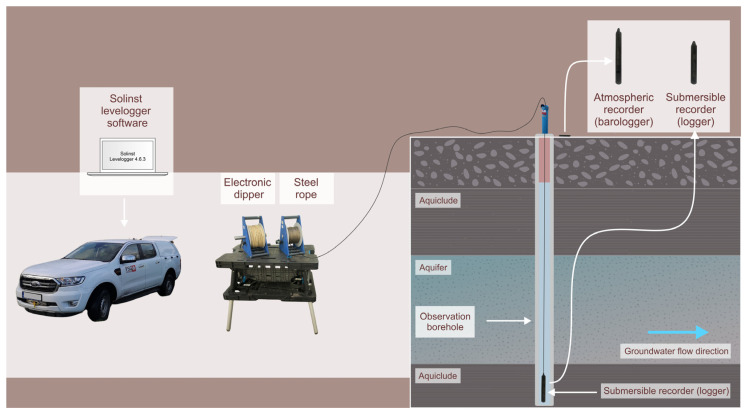
Field setup of temperature logging with manual recorders.

The groundwater level in the boreholes was always measured with an electronic dipper on a measuring tape. Usually, the measurements started about 1 m below the groundwater table in the borehole and the submersible recorder was left in this position for about 10 min for the sake of temperature balancing between the recorder and groundwater. Thereafter, the Levelogger 5 recorder was lowered manually into the borehole on a steel wire at an average speed of approximately 4 (m/min), while the sampling frequency was usually set to four seconds which provides a single point measurement of about 0.4–0.6 m. In this way, the depth-dependent temperature measurements yielded nearly continuous results, allowing for detailed analysis. The Barologger 5, recording atmospheric temperature and pressure, was placed at the surface close to the borehole. The field setup of the equipment used for the manual temperature logging is shown in Figure 2.

### 2.4. OF DTS Technology, Calibration and Measurements

Theoretical knowledge regarding the principles of OF DTS measurements is described below in rather a general manner, while comprehensive information is provided in other specific publications [27,28,29]. At the core of most DTS units, termed the “interrogator”, is a pulsed laser source coupled to the optical fibre in both ends. The laser light is propagated along its entire length. The glass OF is thus a passive sensor, packaged within a cable construction designed to withstand the temperature and mechanical constraints of any given application. All along the fibre, the laser light undergoes multiple scattering processes. Some portion of the light is scattered back and returned to the interrogator [30]. The light returning from any given scattering location along the OF contains information on the physical conditions close to the scattering location. Through time-resolved detection of the scattering, it is possible to build up a profile of the scattered light along the entire fibre, forming the basis of the distributed sensor technology [27].

Three types of scattered light spectrum in an optical fibre have been identified: Rayleigh, Brillouin and Raman. Rayleigh scattering is an elastic scattering natural inhomogeneities within glass and is used for distributed acoustic sensing (DAS) in applications such as security [31], geophysical studies of the subsurface [32] and transport infrastructure monitoring [33]. Raman and Brillouin light are generated by a non-linear interaction between the light source and the glass in the optical fibre, giving rise to inelastic scattering. Brillouin scattering has thus been used for distributed strain scattering (DSS) [34,35], while Raman scattering is used for distributed temperature sensing (DTS) [36]. A DTS interrogator measures the intensity of the back-scattered light at two distinct wavelengths produced during the Raman scattering process, termed Stokes and anti-Stokes. The intensity of the anti-Stokes wavelength produced from the scattering at any given location is dependent on the temperature. In this way, the optical cable becomes a passive temperature sensor that can be used in several contexts, including geology, hydrogeology and geothermal energy [28,37]. Several methods exist for calibrating DTS data. The calibration algorithms used vary and are chosen depending on factors including practicality, costs, the required accuracy and the expected temperature variation [38]. A common element in any calibration method is to have one or several sections of the fibre immersed in calibration baths at a constant and uniform temperature, where the length of fibre immersed should exceed the spatial resolution of the instrument. Calibration algorithms also depend on the fibre configuration and can be divided into three categories. The first one is “single”-ended, where a single fibre connected to the interrogator is placed along the test section. This is the simplest solution, which maximizes the test length that can be probed by the interrogator. A disadvantage with the single-ended configuration is that it is often impractical to have a reference bath at the end of the fibre in addition to being close to the interrogator, therefore reducing the achievable accuracy. The correction of step losses in the single-ended configuration is difficult. Such disadvantages are mitigated in the “duplexed, single-ended” method, where the fibre is looped back towards the interrogator at the end of the measurement section, but not connected to it. The returning end is arranged to pass through a reference bath, avoiding the complexity of installing the bath at the end of the reference section. The fact that the duplex single-ended configuration produces two (dependent) measurements at each location also improves the ability to compensate for step losses. The final configuration, “doubled-ended”, an extension of the single-ended duplex where the retuning fibre is connected to a second port on the DTS interrogator, enables the fibre to be illuminated in both directions, resulting in two duplex traces. The double-ended measurement enables the impact of the losses to be reduced even further relative to the duplex configuration.

**Figure 3:**
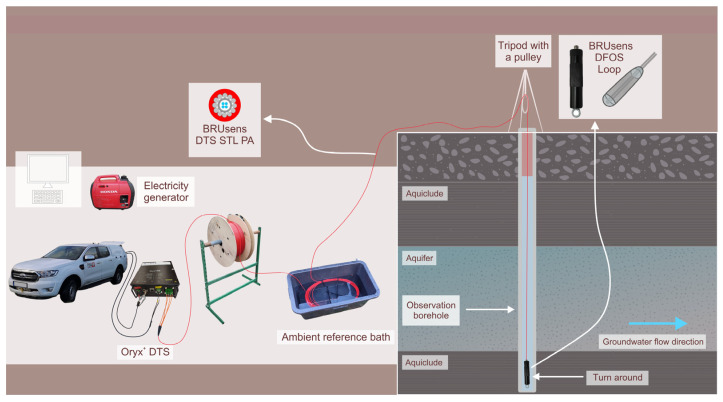
Field setup of OF DTS.

For our measurements, an Oryx^+^ DTS interrogator unit (Sensornet Ltd., Watford, UK) was used [39]. The measurements were carried out between November 2023 and January 2024. For all the measurements, an approximately 400 m long multimode fibre optic cable (Solifos, model BRUsens DTS STL PA, Windisch, Switzerland) was used [40]. The cable contains four fibres that are connected in pairs at one end using a turnaround, which enables the use of duplex and double-ended measurements. The OF cable was coiled on a drum, which in the field was mounted on a steel rack. The cable was manually lowered down into the borehole with the use of a pulley suspended on a tripod. The field setup of the equipment used for the OF DTS measurements is shown in Figure 3.

**Table 2:** Technical characteristics of manual temperature recorders and OF DTS.

	Automatic Recorders [26]: Levelogger 5 Model 3001 LTM200 Barologger 5 Model 3001LT M1.5	DTS Unit with Optical Cable: Oryx^+^ DTS [39] and OF Cable BRUsens DTS STL PA [40]
Producer	Solinst Canada Ltd., Georgtown, ON, Canada	Sensornet Ltd., Watford, UK and Solifos AG, Windisch, Switzerland
Level sensor	Piezoresistive Silicon with Hastelloy^®^ Sensor	No
T sensor	Platinum Resistance Temperature Detector (RTD)	OF cable BRUsens DTS STL PA
T sensor accuracy	±0.05 °C	±0.5 °C
T sensor resolution	0.003 °C	0.01 °C
T compensation range	0 to +50 °C	−200 to +600 °C depending on cable type
Max T measurement range	200 m b.w.l. *	12,500 m max OF cable length
Operating T	−20 to +80 °C	−40 to +65 °C
Spatial sample spacing	0.4–0.6 (m) **	0.5 (m)
Battery lifetime	10 years (1 reading/minute)	15 years
Barometric compensation	High accuracy, Air only, Barologger	No
Measurement without extra power source	Yes	No

m b.w.l.—metres below water level, measurements in pure water: * max height of water column, ** average value range taking into account average logging speed of 4 (m/min) and sampling frequency of 15 s.

**Figure 4:**
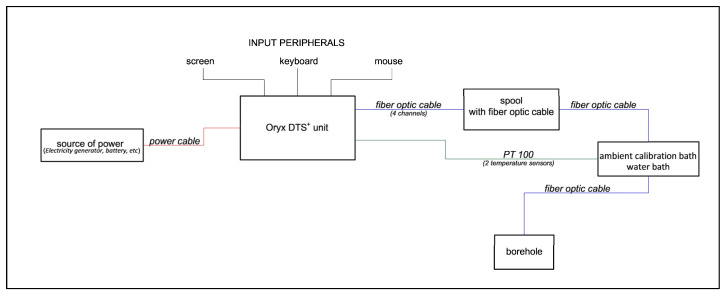
Connection scheme of the devices used for OF DTS measurements.

The Oryx+ DTS device has four channels, into which four fibres of the fan-out ending were connected [39]. Channels 1 (Ch. 1) and 2 (Ch. 2) connect the same optical fibre going downwards and upwards, respectively, through which the laser signal is propagated. Similarly, channels 3 (Ch. 3) and 4 (Ch. 4) connect one and the same fibre of the OF cable [40]. The scheme of the connection of the devices for the OF DTS measurements is illustrated in Figure 4.

**Figure 5:**
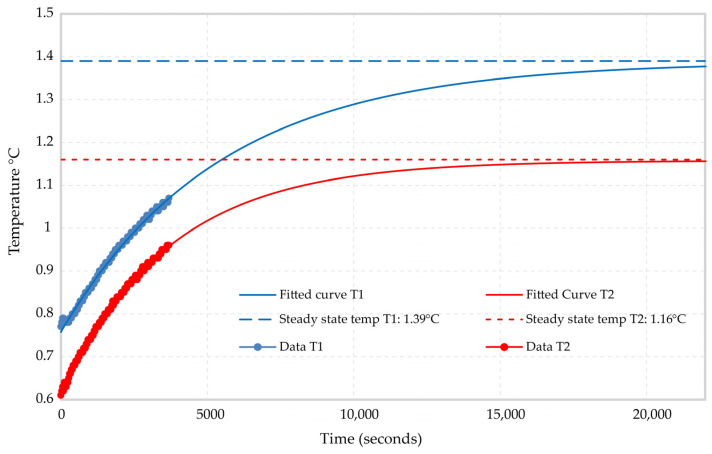
Calculation of correction factors for two PT100s used for calibrating DTS data. The PT100s were submerged in an ice bath while logging the temperature. Curves were fitted to the resulting time series in order to give a correction factor based on the steady-state temperature.

**Figure 6:**
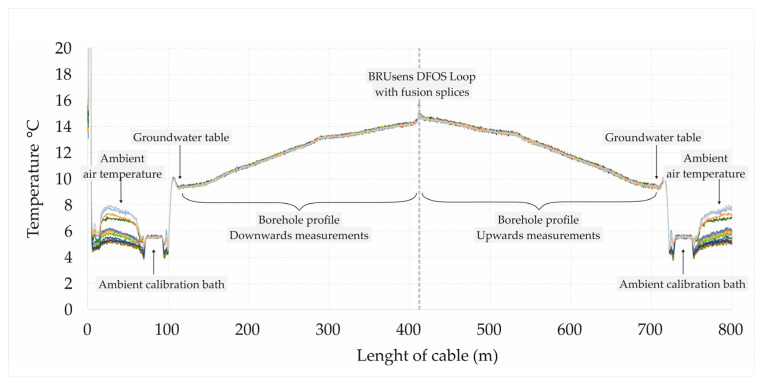
DTS data obtained from channel 4 (Ch. 4) for borehole no. 15 Wambierzyce 18N in the anti-Stokes and Stokes regime are shown by colorful lines. The data covered a time period of about 40 min, explaining the variability in the observed ambient air temperature. The data before and after ~410 m corresponds to Raman backscatter produced by laser pulses traveling downwards and upwards along the cable, respectively.

Calibration of the Oryx+ DTS device is required before each measurement series. This was assured by submerging at least 10 m of the cable in an ambient calibration bath well insulated from all sides and filled with water at a temperature close to the ambient air temperature (Figure 5). Furthermore, the water in the calibration bath was well stirred prior to each measurement to assure a homogenous calibration medium in terms of temperature. In one case, the calibration was completed by burying the cable reference section in snow, showing a very stable temperature during all the measurements. The temperature measurements for the calibration were acquired using two PT100 sensors. It should be noted that the calibration is updated and applied automatically to each acquired dataset using the latest measurements from the PT100s. This is sufficient to ensure that the system is always calibrated, provided that the calibration bath is stable in temperature. In order to compensate for an offset, we observed in the PT100s relative to the Solinst that a correction factor for the DTS temperature calibration was acquired by submerging the PT100s in an ice bath for around one hour while logging the data (see Figure 5). A natural logarithm-based curve was fitted to the resulting time series data to extract the equilibrium readings of each sensor in the ice bath, giving the correction factor for the DTS calibration. Given the calibration from two reference sections (both ends of the cable), the interpreted temperature using the data collected a single-ended duplex configuration is shown in Figure 6.

### 2.5. Factors Influencing Temperature Measurements and Patterns of Depth-Dependent Temperature Curves

In the past, manual temperature logging was conducted for some of the boreholes studied in this research. The obtained temperature referring to the zone below the daily and seasonal variations has shown very similar values for both measurement series. In the case of OF DTS, each measurement was performed about 20 times using all four DTS channels. The measured temperature values were observed with the use of a screen connected to the DTS unit in real time. No essential temperature variations during measurements were observed. Therefore, it was presumed that both methods provide reliable and reproducible results. The temperature values taken into account for further analysis were average values.

The results of the manual temperature logging and DTS measurements were analysed in the context of the depth-dependent temperature features. Usually, the top parts of the temperature logs vary significantly between individual boreholes, depending on such factors as ambient air temperature, temperature and the amount of precipitation, altitude, land use type, year, season and many others. These changes occur within a zone called a zone of daily and seasonal temperature fluctuations, which is usually up to a few metres thick. Below this, a zone of so-called transient or neutral temperatures appears, where the fluctuating subsurface temperature converges and shows a nearly stable value throughout a longer period, for instance over decades. The depth of this zone may differ from location to location depending on several geo- and anthropogenic factors. In the case of a lack of any abnormal thermic tendencies within the subsurface, the zone of neutral or transient temperatures is normally located at a depth of about 15–20 m. At some depths under that zone, an inflection point occurs below which the subsurface temperature depends strictly on the local geothermal gradient [6,41]. The calculation interval that is described below always occurs within this zone.

### 2.6. Calculation Procedures of the Selected Geothermal Parameters

In this research, the heat flow was determined only for the reason of demonstrating the use of both methods and to prove if their result are comparable in Polish conditions. Therefore, it was decided that the calculations could be conducted taking into account appropriate simplifications, omitting conduction and convection, and the vertical and horizontal thermal conductivity of the pipes, cementation and grouting, as well as radiogenic heat due to the decay of unstable isotopes in the lithosphere. The heat flux of the Earth as a conductive component was calculated according to Equation (1).

(1) Q=−K·G

where *Q* is the surface heat flux of the Earth (mW/m^2^) and *K* is the thermal conductivity of the subsurface (mW/m°C), with the minus sign indicating the direction of the upward heat flow, implying heat travelling from the depth towards the surface of the Earth, while the depth from the top of the well is denoted by *H* (m) in metres below ground level (m b.g.l.), implying a positive direction downwards. Thus, *G*(*H*) is the measured depth-dependent geothermal gradient (°C/m).

The deeper parts of the temperature logs showed a direct dependency on depth, i.e., temperature growth with depth. There, the calculation intervals were designated. The geothermal parameters were calculated for the boreholes, showing a thickness of the calculation interval of about 100 m or more. For comparative reasons, an exception was made and calculation intervals of tens of metres were taken into account. The calculations of the geothermal gradient were performed according to Equation (2).

(2) $$G\left(H\right)\;=\;\frac{∆T}{∆H}\;=\;\frac{T_{n}\;-\;T_{0}}{H_{n}-H_{0}}$$

where *G*(*H*) is the depth-dependent geothermal gradient (°C/m), Δ*T* is the temperature change (°C), Δ*H* is the thickness of the geological log of the borehole (m), *T_n_* is the temperature at the bottom of the calculation interval (°C), *T*_0_ is the temperature at the top of the calculation interval (°C), *H_n_* annotates the depth of the bottom of the calculation interval (m b.g.l.) and *H*_0_ is the depth of the top of the calculation interval (m b.g.l.). The calculated average values of the geothermal gradient were used to calculate the average value of the heat flux according to Equation (1). The calculations assumed simplification of the procedures. The thermal conductivity, *K_ef_*, was determined for the individual boreholes, taking into account the diversity of the lithology and thermal properties of the rock types along the calculation interval and was calculated as a weighted mean according to Equation (3):

(3) $$K_{ef\;}=\;\frac{h_{1}\;+\;h_{2}\;+\;\cdots \;h_{n}}{\frac{h_{1}}{K_{1}}\;+\;\frac{h_{2}}{K_{2}}\;+\;\cdots \;\frac{h_{n}}{K_{n}}\;\;}$$

where *K_ef_* is the effective thermal conductivity (mW/m°C) for the calculation interval, *K*_1_, *K*_2_ and *K_n_* are the thermal conductivities of the individual rock types (mW/m°C) and *h*_1_, *h*_2_ and *h_n_* are their thicknesses (m). The effective thermal conductivity values were assumed according to the specification provided in the Earth Energy Designer application [42].

## 3. Results and Discussion

### 3.1. Thermal Regime of the Subsurface

A fundamental premise of all the temperature measurements is that the water column in each studied borehole was under thermodynamic equilibrium with the surrounding geothermal gradient within the rocks and aquifers. This is supported by the fact that all the boreholes were shut in for several months after the previous temperature logging campaigns were performed in 2022, and, in a few cases, in 2023. It has been also assumed that the shut-in time between the manual logging and the OF DTS measurements conducted for the sake of the present research was long enough to provide appropriate results.

The certainty of the OF DTS measurements results was assured by the appropriate calibration of the equipment in the field. Some disadvantages of the continuous manual temperature logging and lowering the submersible unit into the borehole could have been the vertical mixing of groundwater, causing possible perturbation of the measurements. Nevertheless, no clear evidence of such an effect was observed for the obtained results; thus, the possible adverse effect was neglected. No such effect was observed for OF DTS either. The measurements carried out for the studied boreholes allowed us, among other outcomes, to obtain the depth-dependent temperature curves (logs). Each of them is unique and shows the specific temperature variability along its entire length. The changes distinguishable along the temperature logs depend on several factors. Among the natural ones are the lithology and thermal conductivity of the individual rock types, pore water content, the presence of aquifers, aquitards and aquicludes, groundwater flow velocity and its direction, and many others. The evaluation of the patterns of the temperature logs requires the analysis of several effects [43], which will be the subject of separate studies. This paper, however, focuses on proving the suitability of both methods and comparing their results. The shallower parts of temperature logs are normally affected by seasonal temperature variations [44], anthropogenic factors and zones of active thermal energy exchange of natural origin [45,46,47], while in the deeper parts, the overall geothermal gradient dominates the observed behaviour [41]. All the factors are instrumental when assessing the shallow region ground source heat potential.

**Figure 7:**
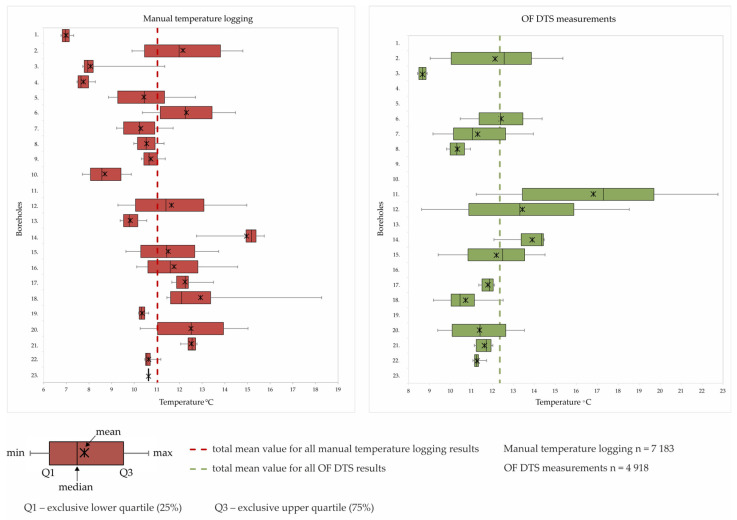
Box plots showing statistical parameters for temperature measurements by manual logging and OF DTS for the individual boreholes.

**Figure 8:**
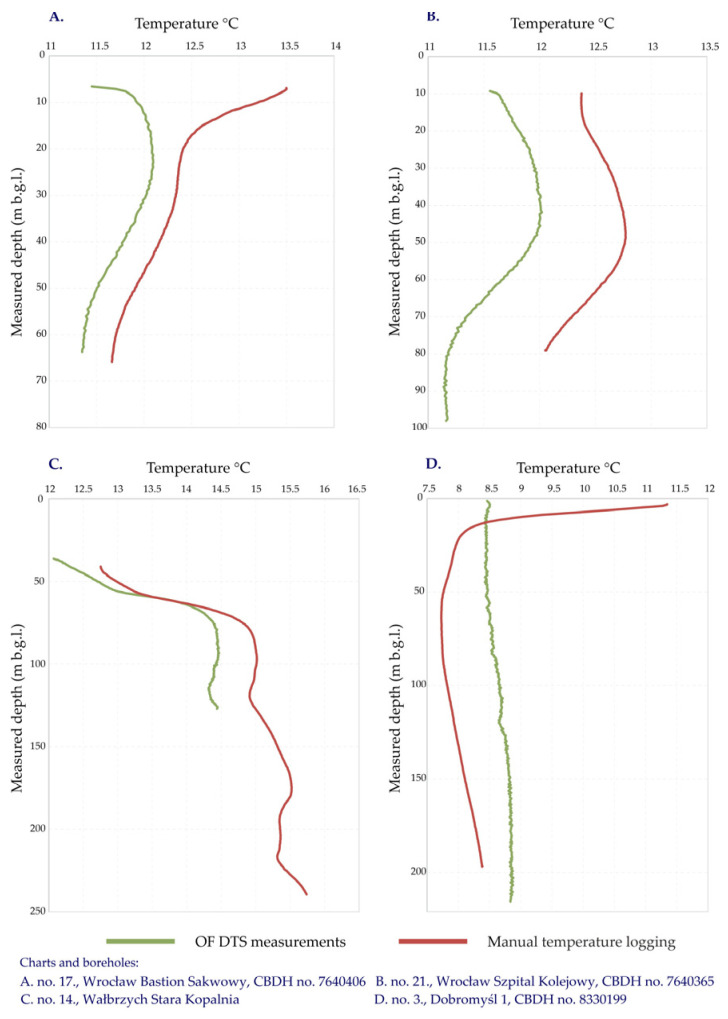
Depth-dependent temperature curves in the selected geological boreholes for manual logging and OF DTS measurements showing strong anthropogenic impact. Since the geothermal signal is overrun, these boreholes were excluded from the assessment of vertical heat flow.

**Figure 9:**
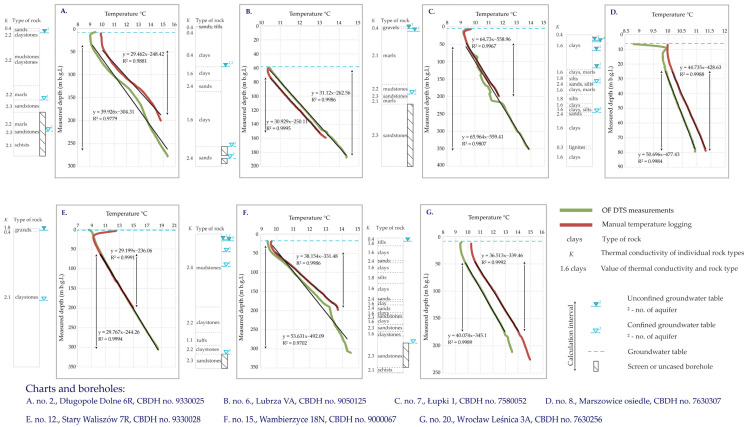
Depth-dependent temperature values with calculation intervals and thermal conductivity values of the rock types for the selected boreholes. Values of thermal conductivities were set up according to Earth Energy Designer application [42]. Values of weighted thermal conductivities for each calculation interval are shown in Table 6.

Two temperature measurement campaigns took place under different weather conditions, i.e., manual temperature logging in autumn, when the ambient air temperature values were well above 0 °C, while the OF DTS measurements were conducted in winter, when the ambient air temperatures were both freezing and above 0 °C. An overview of all the boreholes, the dates of the temperature logging campaigns and the measurement technology used is shown in Table 3. The general characteristics of the shallowest part of the temperature logs for both methods may differ considerably, while the deeper one shows very similar features. These aspects can be observed in Figure 7 and Table 4 and Table 5, showing the statistical parameters of the measurements for the individual boreholes, as well as in Figure 8 and Figure 9, where the depth-dependent temperature curves are shown.

The statistical parameters shown in Table 4 and Table 5 as well as in Figure 7 help to interpret the measurement results. In the case of the manual temperature logging, the total number of obtained and analysed measurements was 7183, while for the OF DTS method it amounted to 150,666. The small spread of data presented in Figure 7 normally refers to the shallow boreholes, while the wider spread refers to the deeper ones. The minimum groundwater temperature was always measured within the zone of seasonal variations and amounted to 8.43 °C for the manual temperature logging and up to 6.77 °C for the OF DTS method for boreholes no. 3 and 1, respectively. The maximum temperature for the manual temperature logging was 15.74 °C for borehole no. 14, while for the OF DTS method it reached 22.76 °C in the case of borehole no. 11. The difference between the maximum measured temperatures for the individual methods is caused by the depth of the measurement, which was usually higher for OF DTS. The range of the measured temperature values was higher for OF DTS than for the manual logging, which amounted to 11.53 °C and 6.84 °C, respectively. It is necessary to underline that the total mean value for all the measurements using the OF DTS method was higher than that obtained using manual logging; these values were 12.35 °C and 11.02, respectively.

### 3.2. Irregular Temperature Patterns

The initial analysis of the temperature logs allowed for the selection of results for further consideration and calculations. At the shallow and sometimes even the medium depths, the observed temperature values were, to a large extent, dictated by external anthropogenic factors including the influence of urban heat islands, post-industrial effects and the presence of diverse infrastructure. Other observed irregularities of the subsurface temperature result from, for example, artesian hydrological groundwater flow. A group of 16 boreholes, namely no. 1, 3, 4, 5, 9, 10, 11, 13, 14, 16, 17, 18, 19, 21, 22 and 23, had irregular patterns and did not show a clear temperature trend depending solely on the geothermal gradient; thus, for these locations, it was not possible to set a calibration interval allowing for the calculation of the geothermal parameters (Table 1 and Table 3). These boreholes were disregarded in further quantitative analysis. The selected examples of the excluded temperature logs for the four locations are illustrated in Figure 8.

The measurements in several boreholes yielded temperature logs with strong features, indicating anthropogenic influence even to a depth of tens of metres. This is evident when comparing the temperature profiles in urban areas to those obtained for suburban and rural regions. Typical examples are the boreholes located within the city centre of Wrocław (boreholes no. 17, 18, 21 and 22), where the temperature logs of the upper tens of metres were dominated by the urban heat island and the man-made underground infrastructure. Such temperature irregularities have been already confirmed by earlier research [48,49,50]; therefore, these are presumed to be a long-lasting effect, which is known to have occurred in other locations as well [51]. The two selected boreholes illustrating the anthropogenic influence on the temperature regime of the subsurface in the city of Wrocław are shown in Figure 8A,B.

Another strongly irregular pattern may be seen in borehole no. 14, located in the vicinity of a former mine shaft and not previously recognised to have been affected by post-mining processes (Figure 8C). It must be underlined that this borehole is much deeper than the maximum measured depth; thus, its thermal profile is not fully known. An individual and potentially interesting case is borehole no. 3, which remained occasionally under artesian pressure. During the manual temperature logging, the groundwater head in the borehole was under-confined; however, this was not artesian pressure, while during the OF DTS measurements, the inner pressure in the aquifer was higher and the groundwater was flowing out of the borehole. This means the manual temperature logging was performed under steady-state thermodynamic conditions, while the OF DTS measurements were obtained under transient ones. Figure 8D. displays the difference in the temperature patterns of the same borehole under these different conditions.

Irregularities of other types can be observed in boreholes no. 2, 6, 7, 12, 15 and 20 (Figure 9) within the geothermal gradient temperature-dependent zone. In fact, it is difficult to distinguish their background and sources. In the case of borehole no. 15 (Figure 9F), the increase in temperature at a depth of about 190 m might be caused by the change of lithology, while in the case of borehole no. 20 (Figure 9G), the temperature disturbance at a depth of about 180 m may have been caused by groundwater flow into the screen. Several irregularities were detected for borehole no. 7 (Figure 9C); however, these are recognizable only by the OF DTS method and cannot be observed on the log for manual logging; therefore, their interpretation is complex.

### 3.3. Calculation of the Selected Geothermal Parameters

The prerequisite for the temperature log to allow for the calculation of the geothermal parameters is its relatively strong and undisturbed dependence on the geothermal gradient observed below the inflection point, allowing for the designation of a calculation interval showing a thickness of about 100 m or more, with the exception of boreholes no. 6 and 8. Such patterns were observed for seven boreholes only, namely boreholes no. 2, 6, 7, 8, 12, 15 and 20 for both the manual temperature logging and OF DTS method. Figure 9 shows the temperature logs for both types of measurements together with a petrological lithology assessment of the individual rock types for the calculation of the effective thermal conductivity. Here, a calculation interval is chosen when the depth-dependent temperature curve is dominated by the overall geothermal gradient. The conductivity of each layer and the estimated conductivity of the mixture from the calculation interval is used in Table 6 to estimate the overall heat flux. The main reason for the discrepancies in the geothermal gradient, effective thermal conductivity and heat flux values shown in Table 6 between the two methods applied in this research are due to the different calculation intervals for each of them. Those are usually shorter for the manual logging than for the OF DTS measurements, which is shown in Figure 9. The different thickness of the calculation intervals ensures that different sections of the lithological profile, and thus, different types of rocks, are taken into considerations for further calculations.

Each borehole shown in Figure 9 reflects some differences in terms of the geological, hydrogeological and climatic settings, which in effect influence the obtained results. Some of them, e.g., boreholes no. 6, 8, 12 and 20, shown in Figure 9B, 9D, 9E and 9G, respectively, show patterns very strongly related to the thermal gradient of nearly the entire measured length. For most boreholes, the temperature curve for the manual logging and the OF DTS measurement does not coincide with each other, except for borehole no. 12 (Figure 9E). The last one is located in a thick and relatively homogenous claystone series. The observed irregularities certainly depend on the thermal conductivity of the individual types of rocks. In addition, a significant impact of the aquifers and aquicludes could be also observed. It is necessary to stress that the shallowest parts of the temperature curves often show differences in temperature value due to the fact that the manual logging was conducted in autumn and the OF DTS measurements in winter. The position of the temperature vs. depth curves could be different depending on the measurement method, which may be also to some extent explained by the measurement season.

The minimum thickness of the calculation interval amounts to 54.53 and 55.08 m for the manual temperature logging and OF DTS methods, respectively, for borehole no. 8. In the case of borehole no. 6, the thickness of the calculation interval was 79.52 m for the manual temperature logging and 124.44 for the OF DTS method. Those measurements allowed for further calculations in order to compare the calculation results of both methods. The maximum thickness of the calculation interval for the manual temperature logging is 159.42 m, while for the OF DTS method it is 317.72 m. The values of the geothermal parameters calculated for the two measuring methods show relatively small differences, for which the main reason may be the maximum measurement depth. This is usually much higher for the OF DTS method than for manual temperature logging due to the limitation of the maximum pressure of the water column for the submersible recorder. The differences between the thickness of the calculation interval, such as, for example, in the case of borehole 15, may cause variations between the values of the calculated parameters for the two methods. According to the authors’ opinion, the most reliable values of the calculated thermal parameters are yielded for the deepest and thickest calculation intervals. The calculation values of the geothermal parameters, including the geothermal gradient and heat flux achieved under the terms of the present study, reflect to a high extent the results of previous research both on the scale of Europe [52] and in Poland [53,54,55,56,57], as well as on the regional scale [58]. This is an essential observation in terms of the discussion of the results, as the earlier research took into account much thicker calculation intervals, usually covering the total accessible depth of the borehole.

## 4. Conclusions

Optical Fibre Distributed temperature sensing and manual temperature measurements was performed for 23 boreholes in south-western Poland in autumn 2023 and winter 2023–2024, respectively. This work was conducted as a stepping stone towards developing a regional assessment of the geothermal resource potential. Such assessments may prove useful when planning for the extraction of reliable, local and renewable heat in the development of a carbon-neutral energy system. It is well established that extractable heat comes from both geothermal gradients and hydrological processes and to some extent from anthropogenic sources (e.g., densely populated areas and mining activities). When observing the different temperature profiles along the wells, it may be seen that the observed geothermal trends in the shallow segments were overrun by both anthropogenic heat sources and artesian underground water flow.

When developing a regional geothermal heat flow map from the Earth, data to calculate the geothermal gradients could only be extracted from the selected deeper segments of the boreholes. A petrophysical assessment of the rocks was performed to obtain the vertical thermal conductivities for the individual rock types. By combining the geothermal gradient and effective thermal conductivity, the heat flux was estimated for eight boreholes with both manual temperature logging and OF DST measurements. The published dataset is a key element in determining the local thermal patterns as well as the geothermal potential.

Both measurement methods used in the present study show individual advantages and disadvantages. Manual temperature logging can provide very precise results showing high resolution when the submersible recorder is lowered down a borehole with a nearly constant speed, which might be a solution to some technical difficulties. A severe limitation of this method is the resistance of the sensor to the water column pressure in the borehole. The OF DTS method provides reliable measurement results and can be used in boreholes showing depth of up to several hundred metres. Both methods are suitable for applications connected with geothermal research and the further calculation of geothermal parameters necessary to locate geothermal installations and optimize their parameters. Those can be also used to identify zones of intensified groundwater flow, groundwater in-rush into the wells, fractured and fissured zones and many others. Since OF DTS systems have the advantage that they can create new temperature depth profiles on a continuous basis without moving the cable, long-term groundwater studies may be able to reveal temporal/seasonal variability and the impact of slow subtle changes due to the anthropogenic effect and monitoring climate change. The continuous measurement capability of OF DTS may also allow for the capture of transient effects that may be missed during a manual temperature log. DTS instruments are high cost, multi-location, long-term studies using DTS, which could be facilitated by the development of lower cost instrumentation. Groundwater OF DTS studies where the temperature differential between the borehole and inflows is low could benefit from “active” DTS, whereby heat is applied through the inclusion of an electrical resistance wire in the fibre cable. Future studies will benefit from continuous advances in DTS instrumentation performance with respect to increased spatial resolution and sensitivity.

## Figures and Tables

**Table 3:** Summary of manual temperature logging and OF DTS measurements results.

# *	CBDH ID No. **	Manual T Logging					OF DTS				
		Date dd.mm.yyyy	Ambient Air T °C	Ground Water Table Depth (m b.g.l.)	Max Meas. Depth, (m b.g.l.)	T at Max Meas. Depth °C	Date dd.mm.yyyy	Ambient Air T °C	Ground Water Table Depth (m b.g.l.)	Max Meas. Depth (m b.g.l.)	T at Max Meas. Depth °C
1.	8310064	19.09.2023	17.53	7.97	52.09	7.33	n.a.	n.a.	n.a.	n.a.	n.a.
2.	9330025	19.10.2023	13.26	8.69	199.63	14.80	17.01.2024	−2.50	7.61	277.50	15.34
3.	8330199	03.11.2023	9.24	1.75	196.55	8.34	19.12.2023	7.00	0.00	215.50	8.84
4.	8330178	24.10.2023	15.43	27.11	153.44	8.28	n.a.	n.a.	n.a.	n.a.	n.a.
5.	9000064	20.10.2023	17.22	0.62	198.19	12.70	n.a.	n.a.	n.a.	n.a.	n.a.
6.	9050125	03.10.2023	29.90	59.12	192.63	14.47	24.01.2024	9.00	58.93	187.26	14.36
7.	7580052	18.10.2023	17.73	5.44	199.53	11.72	25.01.2024	6.00	5.14	357.90	13.94
8.	7630307	13.09.2023	25.14	6.10	78.74	11.32	12.01.2024	1.00	6.92	79.40	10.94
9.	7630154	13.09.2023	29.92	5.20	74.78	11.37	n.a.	n.a.	n.a.	n.a.	n.a.
10.	8330135	24.10.2023	14.87	6.11	108.99	9.87	n.a.	n.a.	n.a.	n.a.	n.a.
11.	6890081	n.a.	n.a.	n.a.	n.a.	n.a.	18.12.2023	10.00	26.23	338.50	22.76
12.	9330028	19.10.2023	12.52	1.25	199.17	14.97	17.01.2024	2.50	0.87	305.50	18.52
13.	8670005	25.10.2023	13.58	10.73	94.66	10.37	n.a.	n.a.	n.a.	n.a.	n.a.
14.	n.a.	09.10.2023	9.33	38.80	239.43	15.74	30.01.2024	10.00	36.71	127.00	14.44
15.	9000067	28.10.2023	13.96	16.79	197.11	13.73	23.01.2024	5.50	16.72	310.50	14.51
16.	6890064	10.10.2023	12.18	0.34	149.44	14.08	n.a.	n.a.	n.a.	n.a.	n.a.
17.	7640406	20.09.2023	23.14	5.43	65.88	11.66	26.01.2024	5.80	5.40	63.70	11.35
18.	7641078	28.09.2023	16.26	2.43	58.54	11.44	09.01.2024	−8.30	2.26	58.50	9.92
19.	7630374	28.09.2023	26.07	12.02	54.82	10.64	n.a.	n.a.	n.a.	n.a.	n.a.
20.	7630256	28.09.2023	25.28	10.69	224.89	15.02	05.01.2024	3.50	10.39	211.20	13.52
21.	7640365	20.09.2023	19.67	8.86	79.04	12.05	10.01.2024	−10.00	8.99	98.50	11.13
22.	7641997	20.09.2023	16.07	11.76	88.38	11.17	06.12.2023	4.80	11.50	79.50	11.72
23.	7630295	13.09.2023	27.01	22.15	47.39	10.65	n.a.	n.a.	n.a.	n.a.	n.a.

* Borehole numbering according to Figure 1, Table 1; ** CBDH ID no.—unique no. in the HYDRO Bank [24] database; T—temperature; n.a.—not available.

**Table 4:** Statistical parameters characterizing temperature values °C measured in the studied boreholes with manual temperature logging method.

# *	CBDH ID No. **	Max Measured Depth (m b.g.l.)	n ***	Min	Max	Mean	Median	Range	Q1	Q3	IQR	Standard Deviation
1.	8310064	52.09	159	6.77	7.33	6.99	6.97	0.56	6.82	6.97	0.15	0.17
2.	9330025	199.63	361	9.90	14.80	12.15	11.98	4.89	10.46	11.98	1.53	1.69
3.	8330199	196.55	379	7.73	11.35	8.07	7.95	3.62	7.79	7.95	0.16	0.51
4.	8330178	153.44	237	7.47	8.28	7.75	7.65	0.81	7.52	7.65	0.13	0.26
5.	9000064	198.19	385	8.86	12.71	10.42	10.45	3.84	9.27	10.45	1.18	1.18
6.	9050125	192.63	524	10.36	14.47	12.30	12.26	4.11	11.15	12.26	1.11	1.29
7.	7580052	199.53	727	9.22	11.72	10.29	10.23	2.49	9.53	10.23	0.70	0.79
8.	7630307	78.74	263	9.98	11.32	10.55	10.53	1.34	10.14	10.53	0.39	0.42
9.	7630154	74.78	248	10.32	11.37	10.73	10.65	1.05	10.42	10.65	0.23	0.34
10.	8330135	108.99	382	7.71	9.87	8.70	8.57	2.16	8.06	8.57	0.51	0.69
11.	6890081	n.a.	n.a.	n.a.	n.a.	n.a.	n.a.	n.a.	n.a.	n.a.	n.a.	n.a.
12.	9330028	199.17	427	9.28	14.97	11.64	11.40	5.69	10.06	11.40	1.34	1.73
13.	8670005	94.66	311	9.38	10.55	9.82	9.78	1.17	9.53	9.78	0.25	0.33
14.	n.a.	239.43	365	12.75	15.74	14.96	15.18	2.99	14.94	15.18	0.24	0.72
15.	9000067	197.11	357	9.63	13.73	11.50	11.43	4.10	10.29	11.43	1.15	1.32
16.	6890064	149.44	276	10.11	14.56	11.76	11.59	4.45	10.60	11.59	0.99	1.25
17.	7640406	65.88	229	11.66	13.50	12.24	12.25	1.84	11.87	12.25	0.38	0.45
18.	7641078	58.54	218	11.44	18.28	12.93	12.09	6.84	11.60	12.09	0.49	1.92
19.	7630374	54.82	158	10.20	10.64	10.35	10.31	0.44	10.22	10.31	0.09	0.14
20.	7630256	224.89	525	10.26	15.02	12.50	12.52	4.76	11.03	12.52	1.49	1.54
21.	7640365	79.04	265	12.05	12.77	12.52	12.55	0.72	12.38	12.55	0.17	0.20
22.	7641997	88.38	286	10.46	11.17	10.64	10.56	0.71	10.50	10.56	0.06	0.20
23.	7630295	47.39	101	10.61	10.67	10.63	10.63	0.06	10.61	10.63	0.01	0.02

* Borehole numbering according to Figure 1, Table 1; ** CBDH ID no.—unique no. in the HYDRO Bank [24] database; *** n is the number of measurements per borehole, taking into account average speed of logging 4 (m/sec) and sampling frequency 4 sec; Q1—exclusive lower quartile (25%), Q3—exclusive upper quartile (75%); IQR—interquartile range; n.a.—not available; total n = 7183.

**Table 5:** Statistical parameters characterizing temperature values in °C measured in the studied boreholes with OF DTS method.

# *	CBDH ID No. **	Max Measured Depth (m b.g.l.)	n ***	Min	Max	Mean	Median	Range	Q1	Q3	IQR	Standard Deviation
1.	8310064	n.a.	n.a.	n.a.	n.a.	n.a.	n.a.	n.a.	n.a.	n.a.	n.a.	n.a.
2.	9330025	277.50	12,210	9.03	15.36	12.12	12.56	6.33	10.03	13.87	3.84	2.05
3.	8330199	215.50	4310	8.43	8.88	8.66	8.67	0.46	8.49	8.83	0.34	0.16
4.	8330178	n.a.	n.a.	n.a.	n.a.	n.a.	n.a.	n.a.	n.a.	n.a.	n.a.	n.a.
5.	9000064	n.a.	n.a.	n.a.	n.a.	n.a.	n.a.	n.a.	n.a.	n.a.	n.a.	n.a.
6.	9050125	187.26	12,734	10.47	14.37	12.41	12.38	3.90	11.36	13.46	2.10	1.17
7.	7580052	357.90	28,632	9.16	13.95	11.29	11.05	4.79	10.14	12.63	2.48	1.47
8.	7630307	79.40	4764	9.81	10.95	10.33	10.30	1.14	9.98	10.67	0.69	0.37
9.	7630154	n.a.	n.a.	n.a.	n.a.	n.a.	n.a.	n.a.	n.a.	n.a.	n.a.	n.a.
10.	8330135	n.a.	n.a.	n.a.	n.a.	n.a.	n.a.	n.a.	n.a.	n.a.	n.a.	n.a.
11.	6890081	338.50	3047	11.23	22.76	16.81	17.29	11.53	13.43	19.70	6.28	3.53
12.	9330028	305.50	28,106	8.62	18.52	13.43	13.31	9.90	10.87	15.89	5.01	2.85
13.	8670005	n.a.	n.a.	n.a.	n.a.	n.a.	n.a.	n.a.	n.a.	n.a.	n.a.	n.a.
14.	n.a.	127.00	10,160	12.07	14.47	13.90	14.35	2.40	13.37	14.43	1.06	0.75
15.	9000067	310.50	28,140	9.41	14.51	12.19	12.48	5.10	10.83	13.54	2.71	1.58
16.	6890064	n.a.	n.a.	n.a.	n.a.	n.a.	n.a.	n.a.	n.a.	n.a.	n.a.	n.a.
17.	7640406	63.70	2038	11.35	12.10	11.78	11.85	0.75	11.50	12.04	0.54	0.27
18.	7641078	58.50	4680	9.18	12.51	10.72	10.45	3.33	10.03	11.15	1.13	0.83
19.	7630374	n.a.	n.a.	n.a.	n.a.	n.a.	n.a.	n.a.	n.a.	n.a.	n.a.	n.a.
20.	7630256	211.20	10,138	9.39	13.52	11.38	11.41	4.13	10.08	12.64	2.56	1.35
21.	7640365	98.50	4531	11.13	12.02	11.61	11.70	0.89	11.23	11.94	0.71	0.33
22.	7641997	79.50	477	11.07	11.72	11.27	11.23	0.65	11.14	11.33	0.19	0.15
23.	7630295	n.a.	n.a.	n.a.	n.a.	n.a.	n.a.	n.a.	n.a.	n.a.	n.a.	n.a.

* Borehole numbering according to Figure 1, Table 1; ** CBDH ID no.—unique no. in the HYDRO Bank [24] database; *** n is the number of measurements carried at every 0.5 m for each borehole between water level and max. measured depth for selected channel(s) and measurement series, Q1—exclusive lower quartile (25%), Q3—exclusive upper quartile (75%); IQR—interquartile range; n.a.—not available; total n = 150,666.

**Table 6:** Calculation results of major geothermal parameters for the selected boreholes for manual temperature logging and OF DTS measurements.

# *	CBDH No. **	Calculation Interval:			Temperature Within Calculation Interval °C			Geothermal Gradient G (°C/100 m)	Effective Thermal Conductivity K_ef_ (W/m°C)	Heat Flux Q (mW/m^2^)
		Top (m b.g.l.)	Bottom (m b.g.l.)	Thickness (m)	at the Top	at the Bottom	Difference Bottom–Top			
Manual temperature logging										
2.	9050125	40.71	199.63	158.93	10.06	14.80	4.74	2.98	1.58	47.25
6.	9050125	73.32	152.84	79.52	10.49	13.02	2.53	3.18	1.72	54.48
7.	7580052	40.11	199.53	159.42	9.37	11.72	2.35	1.47	2.12	31.26
8.	7630307	24.21	78.74	54.53	10.14	11.32	1.17	2.15	1.44	30.97
12.	9330028	60.75	199.17	138.42	10.24	14.97	4.73	3.42	2.10	71.75
15.	9000067	50.12	197.11	146.99	9.90	13.70	3.80	2.59	2.39	61.77
20.	7630256	44.13	174.66	130.53	10.55	14.04	3.49	2.67	1.81	48.34
OF DTS measurements										
2.	9330025	40.35	275.46	235.11	9.21	15.33	6.12	2.60	2.03	52.75
6.	9050125	60.78	185.22	124.44	10.48	14.35	3.87	3.11	1.73	53.70
7.	7580052	40.18	357.90	317.72	9.36	13.94	4.58	1.44	2.20	31.72
8.	7630307	24.32	79.40	55.08	9.91	10.94	1.03	1.87	1.44	26.99
12.	9330028	60.20	302.96	242.76	10.35	18.49	8.14	3.35	2.10	70.44
15.	9000067	50.41	301.83	251.43	9.73	14.28	4.55	1.81	2.01	36.29
20.	7630256	48.51	177.54	129.03	9.81	12.95	3.13	2.43	1.81	44.02

* Borehole numbering according to Figure 1, Table 1; ** CBDH no.—unique number in the HYDRO Bank [24] database.

## Data Availability

The data presented in this study are available on request from the corresponding authors.
